# A Case of Idiopathic Non-Cirrhotic Portal Hypertension in a Patient With a Left Ventricular Assist Device

**DOI:** 10.1177/2324709619878337

**Published:** 2019-09-25

**Authors:** Shana Kothari, Michael Kalinowski, Natasha Shah, Hareth Raddawi

**Affiliations:** 1 University of Illinois at Chicago/Advocate Christ Medical Center, Oak Lawn, IL, USA; 2 Advocate Lutheran General, Park Ridge, IL, USA; 3 Advocate Christ Medical Center, Oak Lawn, IL, USA

**Keywords:** non-cirrhotic portal hypertension, hepatic venous pressure gradient, endoscopy, left ventricular assist device

## Abstract

Idiopathic non-cirrhotic portal hypertension is a rare diagnosis caused by an unknown etiology with elevated intrahepatic portal pressures in the absence of underlying liver disease. We present a unique case of a 57-year-old male with a left ventricular assist device and preserved right ventricular function that was found to have an elevated hepatic venous pressure gradient and sequelae of portal hypertension without underlying liver disease. There is limited treatment available as management is primarily aimed toward preventing complications of the disease. This case highlights the need for further investigative research of this disease entity and its pathogenesis.

## Introduction

Idiopathic non-cirrhotic portal hypertension (INCPH) is a diagnosis of exclusion with increased portal venous pressure without cirrhosis, hepatoportal flow obstruction, splanchnic venous thrombosis, and other causes of liver disease.^1^ INCPH accounts for 3% to 5% of portal hypertension (PH) cases and 14% to 27% of non-cirrhotic PH cases.^2^ In Western populations, it predominantly affects males with a median age of 40 years; higher incidence at a younger age is seen in Eastern countries presumed to be due to socioeconomic disadvantages and poor living conditions.^1,3^

INCPH presents as complications of PH including variceal bleeding, ascites, portal vein thrombosis, and hepatic encephalopathy.^3^ The underlying pathogenesis remains unclear without specific diagnostic testing. An extensive workup is recommended including laboratory testing, hepatic imaging studies, and a liver biopsy to rule out underlying liver disease. INCPH is a diagnosis of exclusion.

Our case demonstrates the necessity of a thorough workup and the difficulties that occur in managing sequelae of INCPH. Unfortunately, because of unclear pathogenesis, it is difficult to treat, and overall prognosis is poor in the setting of liver failure. To our knowledge, this is the first case of INCPH in a patient with a left ventricular assist device with preserved right ventricular (RV) function.

## Case Presentation

**Figure 1.**
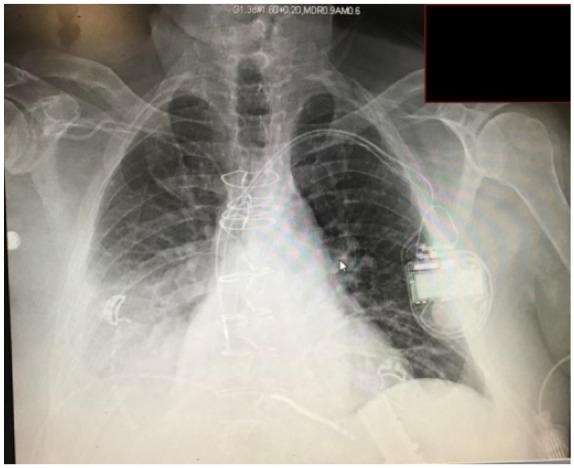
Chest X-ray shows unilateral opacification in right lower lobe.

**Figure 2.**
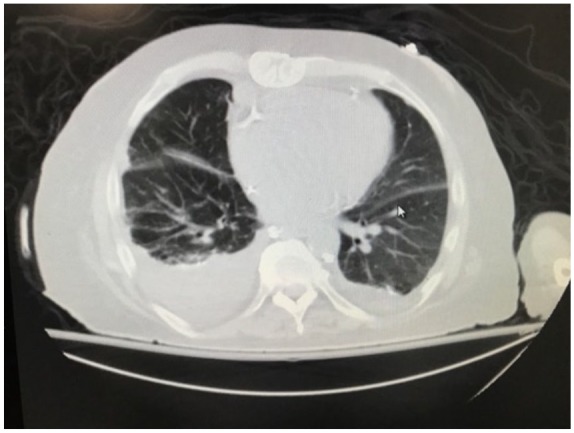
Computed tomography scan of chest showed findings consistent with right-sided pleural effusion.

**Figure 3.**
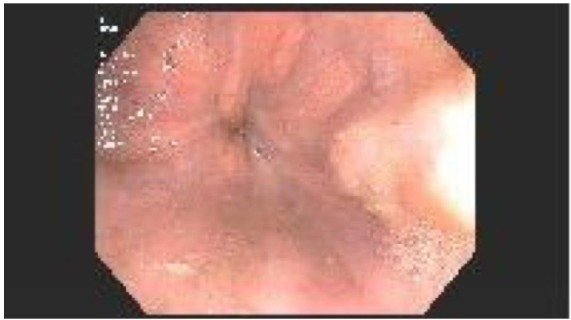
Enteroscopy reveals grade 1 esophageal varices.

A 57-year-old male with ischemic cardiomyopathy and left ventricular assist device presented with dyspnea and melena. Examination was notable for decreased breath sounds. Laboratory tests were significant for anemia (hemoglobin 7.4 g/dL) and transaminitis (aspartate aminotransferase 456 units/L and alanine aminotransferase 528 units/L). Chest X-ray (Figure 1) and computed tomography scan (Figure 2) showed right-sided pleural effusion; thoracentesis revealed transudative fluid. The patient was treated with diuretics and underwent right and left cardiac catheterization, which showed normal pulmonary wedge pressure and RV function with right atrial pressure of 6 mm Hg, right systolic ventricular pressure of 31 mm Hg, and mean pulmonary artery pressure of 22 mm Hg. To evaluate the anemia and melena, the patient underwent colonoscopy and enteroscopy revealing ileal and colonic arteriovenous malformations that were treated and grade 1 esophageal varices (Figure 3). The varices were suspected to be due to chronic liver disease secondary to passive congestion from underlying heart disease. The patient was stabilized and discharged.

**Figure 4.**
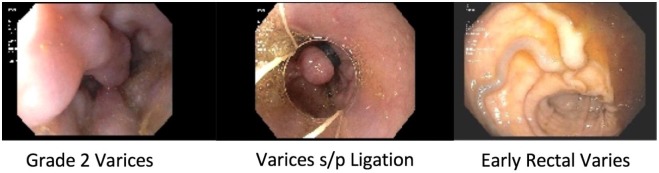
In the distal esophagus, there were 2 columns of grade 2 esophageal varices (A), one of which had a purple blush to it, suggestive of high-risk stigmata. The varices were banded (B). Colonoscopy showed early rectal varices (C).

**Figure 5.**
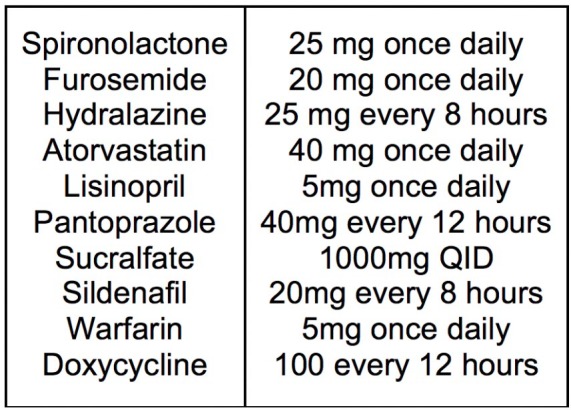
Home medication list.

After 3 months, despite cardiac optimization, the patient developed recurrent pleural fluid accumulation and melena. Repeat upper and lower endoscopy revealed grade 2 distal esophageal varices that were banded, portal hypertensive gastropathy, and rectal varices, suspicious for worsening PH (Figure 4). Repeat cardiac evaluation demonstrated intact RV function. Abdominal ultrasound showed coarsened heterogeneous echotexture of the liver, moderate ascites, and splenomegaly. The transaminases normalized since previous admission indicating rise was likely secondary to fluid overload. Hepatic serologies for underlying diseases such as primary biliary cirrhosis, autoimmune and chronic hepatitis, Wilson’s disease, and hemochromatosis were sent and were all negative. Liver and portal vein vascular duplex revealed patency in the inferior vena cava, portal vein, and right and middle hepatic veins. Medications were reviewed, and no new medications had been started or identified as hepatotoxic (Figure 5).

**Figure 6.**
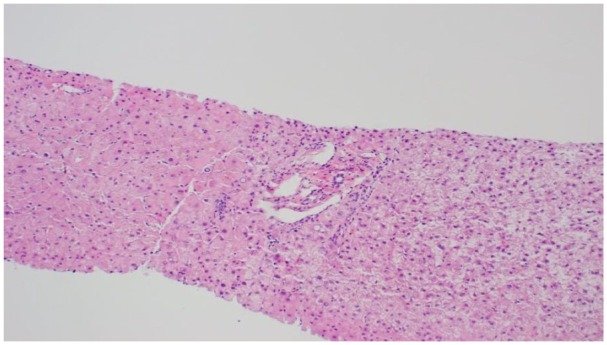
Liver biopsy with normal liver tissue, no evidence of congestive hepatopathy or cirrhosis.

Given suspected cirrhosis without clear etiology, the patient underwent transjugular liver biopsy. The hepatic venous wedge pressure was 21 mm Hg, free hepatic venous pressure was 7 mm Hg, and hepatic venous pressure gradient was 15 mm Hg confirming PH. Liver biopsy revealed normal liver tissue without evidence of congestive hepatopathy or cirrhosis (Figure 6). Repeat liver biopsy, 3 months later, revealed focal lobular inflammation but no evidence of cirrhosis or fibrosis. The etiology of PH remained unclear, and his clinical condition continued deteriorating. The patient developed recurrent ascites, right-sided pleural effusions secondary to likely hepatic hydrothorax (rather than presumed pleural effusion secondary to heart disease), hepatorenal syndrome, variceal bleeding, and encephalopathy. Due to contraindications (heart failure and hepatic encephalopathy), the patient was not a candidate for transjugular intrahepatic portosystemic shunt (TIPS). The patient was transferred to a university center and deemed not a liver or heart transplant candidate, given ongoing sepsis secondary to spontaneous bacterial peritonitis and overall comorbidities. The patient, unfortunately, after suffering from over a year of complications, passed away.

## Discussion

The etiology of INCPH remains unknown; however, literature reports associations with immunological disorders, acute or chronic infections, medications and toxins, genetic disorders, and thrombophilia.^1,2^ These conditions are thought to cause portal venopathy from thrombosis or obliteration due to hypercoagulability, vascular remodeling, endothelial injury, or autoimmune injury from immune complex deposition, autoantibodies, or activated T-cells.^4^

Similar to our patient, the most common presenting sign of INCPH is gastric or esophageal variceal bleeding with preserved liver function.^5^ Due to intact hepatic function, the prognosis of variceal bleeding is improved; acute encephalopathy is a rare complication.^2,5^ Splenomegaly and ascites are found in approximately 95% and 50% of cases, and correlates with poor prognosis. Portal vein thrombosis is relatively common and associated with 13% to 46% of cases.^1-3^

The diagnosis of INCPH has no widely accepted criteria, is underdiagnosed, and commonly misdiagnosed as cirrhosis.^1,2,6^ Liver function tests are typically normal, hepatic and portal veins are unobstructed, and hepatic venous pressure gradient is elevated.^3^ Laboratory tests may show anemia and thrombocytopenia, secondary to hypersplenism.^6^ Viral hepatitis, alcoholic and nonalcoholic steatohepatitis, autoimmune hepatitis, hemochromatosis, Wilson’s disease, and primary biliary cirrhosis must be ruled out via serology and liver biopsy.^1,2,7^ Histological findings are subtle and can be missed, but include dilated sinusoids, fibrotic degeneration of the venous wall, and dense portal fibrosis.

In our case, the patient’s heart failure masked the initial presentation of PH, which was the recurrent pleural fluid accumulation due to hepatic hydrothorax.^8^ This is a rare complication of liver cirrhosis and occurs in 5% to 10% of cirrhotics with only few incidences in INCPH.^9^

The mainstay treatment is to perform TIPS; however, this was contraindicated in our patient given his hepatic encephalopathy and heart failure. Placement of chest tubes in these patients should be avoided as it results in massive protein and electrolyte depletion, infection, renal failure, and bleeding.

INCPH is usually more benign than cirrhotic PH, given preserved liver function. Management is aimed at preventing disease sequelae. Liver transplant is deferred until severe disease progression, although posttransplant outcome data are limited. Whether liver transplant prevents recurrence of INCPH is unclear.

INCPH is a rare pathology that is difficult to diagnose and treat, especially in the setting of multiple medical comorbidities. Limited information is understood regarding this disease; only few studies have investigated INCPH pathogenesis, testing, and treatment. This case raises awareness of a rare disease entity and demonstrates the need for further studies to prevent poor outcomes.
